# Chromosomal microarray analysis for prenatal diagnosis of uniparental disomy: a retrospective study

**DOI:** 10.1186/s13039-023-00668-8

**Published:** 2024-01-30

**Authors:** Chenxia Xu, Miaoyuan Li, Tiancai Gu, Fenghua Xie, Yanfang Zhang, Degang Wang, Jianming Peng

**Affiliations:** 1 Prenatal Diagnosis Center, Boai Hospital of Zhongshan, Zhongshan, Guangdong China; 2 Department of Urology, The People’s Hospital of Zhongshan, Zhongshan, Guangdong China; 3 The First School of Clinical Medicine, Jinan University, Guangzhou, Guangdong China; 4 The Second School of Clinical Medicine, Southern Medical University, Guangzhou, Guangdong China

**Keywords:** Chromosomal microarray, Prenatal diagnosis, Regions of homozygosity, SNP arrays, Uniparental disomy

## Abstract

**Background:**

Chromosomal microarray analysis (CMA) is a valuable tool in prenatal diagnosis for the detection of chromosome uniparental disomy (UPD). This retrospective study examines fetuses undergoing invasive prenatal diagnosis through Affymetrix CytoScan 750 K array analysis. We evaluated both chromosome G-banding karyotyping data and CMA results from 2007 cases subjected to amniocentesis.

**Results:**

The detection rate of regions of homozygosity (ROH) ≥ 10 Mb was 1.8% (33/2007), with chromosome 11 being the most frequently implicated (17.1%, 6/33). There were three cases where UPD predicted an abnormal phenotype based on imprinted gene expression.

**Conclusion:**

The integration of UPD detection by CMA offers a more precise approach to prenatal genetic diagnosis. CMA proves effective in identifying ROH and preventing the birth of children affected by imprinting diseases.

## Background

More than a decade ago, conventional chromosomal karyotyping was considered the gold standard for detecting genome-wide chromosomal abnormalities during the prenatal period [24]. In 2012, Wapner et al. conducted a comprehensive prospective study, revealing that Chromosomal Microarray Analysis (CMA) not only identified additional clinically significant cytogenetic information compared to karyotyping but also demonstrated equal efficacy in detecting aneuploidies and unbalanced rearrangements. However, it should be noted that CMA did not identify balanced translocations and triploidies [26]. Karyotype analysis presents challenges due to its labor-intensive and time-consuming nature, limited resolution, and susceptibility to issues such as maternal cell contamination and culture failure. In contrast, CMA utilizes molecular hybridization technology to discern specific genomic variants. This methodology encompasses array-based comparative genomic hybridization and single nucleotide polymorphism (SNP) array techniques. The SNP array is proficient in identifying regions of homozygosity (ROH), unbalanced chromosome translocations, and certain forms of marker chromosomes. The CMA approach, characterized by its high resolution and swift detection capabilities, has garnered escalating interest in the realm of prenatal diagnosis. CMA is a genomic hybridization method that is superior to karyotype analysis, but it cannot detect balanced recombination and is more expensive [22]. However, pregnant women may also benefit from CMA method, which enables the simultaneous detection of copy number variations, ROH and SNPs [19]. There are three scenarios in which ROH appears in the prenatal sample: (a) Indication of UPD: when a ROH of > 10 Mb occurred on a single chromosome of a fetus (at the end of the chromosome, ROH > 5 Mb,not at the end of the chromosome, ROH > 10 Mb), (b) Identity of descent: ROH > 10 Mb appears on multiple chromosomes, indicating a close parental relationship (the closer the relationship, the more ROH regions), (c) Common ancestral markers in populations. One or a few chromosomes with ROH < 5 Mb. Uniparental disomy (UPD) refers to the inheritance of both homologous chromosomes within a chromosome pair from a single parent. Depending on the chromosomal type involved in UPD, it can be categorized into different types: Heterodisomy, where an individual inherits two different homologous chromosomes from the same parent, and isodisomy, where an individual inherits two identical homologous chromosomes from the same parent. Silver-Russell Syndrome resulting from heterodisomy have been documented in the literature [9]. Only when both parents and offspring are tested for CMA can the presence of UPD be confirmed. Conducting CMA solely on the offspring can only reveal the presence of ROH. The determination of UPD can only be confirmed when both parents and the offspring undergo CMA simultaneously. Some genes on chromosomes exhibit parent-specific expression patterns, meaning they are expressed differently depending on whether they are on the paternal or maternal chromosome. UPD can lead to the exclusive expression of genes from one parent, while the genes from the other parent are silenced. This imbalance in gene expression can contribute to disease because some genes may be suppressed while others are overly activated. These genes are known as imprinted genes. Chromosomes 6, 7, 11, 14, 15, and 20 are chromosomes contain imprinted genes [7, 18]. If the UPD event involves a chromosome that harbors imprinted genes, it may result in an imprinting disorder [7]. Therefore, we advocate for the investigation of parent–offspring trios using CMA to detect (ROH on imprinted chromosomes, aiming to ascertain the presence of UPD. The aim of this study is to analyze 2007 fetuses with ROH ≥ 10 Mb, to identify the high frequency chromosomes with ROH, and to prevent the birth of children affected by imprinting diseases by trios-CMA. Analyzing indications for invasive prenatal diagnosis, we found that the highest proportion of indications. Detection of UPD is a useful diagnostic tool for specific imprinting disorders and rare Mendelian diseases caused by excessive homozygosity [1–3, 14, 28]. This retrospective study was conducted on pregnant women who underwent invasive prenatal testing, G-banding karyotyping, and CMA at the Prenatal Diagnostic Center of Boai Hospital, Zhongshan, between September 2019 and August 2022. The study received approval from the Review Board of Zhongshan Boai Hospital (Approval No. KY-2023-004-46), and written informed consent was obtained from all participating patients. Specimens comprised chorionic villus samples, amniotic fluid, and umbilical blood obtained via abdominal amniotic cavity puncture, guided by B-ultrasound. These collected samples were subsequently utilized for both karyotype analysis and CMA detection. According to the guidelines for the interpretation of fetal chromosomal karyotyping analysis [4], cells were harvested and prepared for G-banding before karyotype analysis was performed. The karyotypes were characterized following the guidelines outlined in the International System for Human Cytogenomic Nomenclature 2020. In this study, CMA was performed using an Affymetrix Cytoscan 750 K array microarray chip. This microarray chip is based on an SNP array platform provided by Thermo Fisher Scientific. The analysis followed the recommended guidelines from the manufacturer, Affymetrix, located in the USA. DNA extraction was carried out using the QIAamp DNA Blood Mini Kit, a product of Qiagen situated in Valencia, CA, USA. The extracted DNA underwent a series of sequential processes, including digestion, ligation, amplification, purification, fragmentation, and labeling. Subsequently, the prepared DNA samples were hybridized on microarray chips. The hybridized microarray chips then underwent washing procedures using the Affymetrix GeneChip Fluidics Station 450, manufactured by Affymetrix in Santa Clara, CA, USA. After the washing steps, the microarray chips were scanned using an Affymetrix GeneChip Scanner 3000. To interpret and analyze the acquired data, the Chromosome Analysis Suite (ChAS) v4.1 software was employed. For the purpose of annotation, the GRCh37/hg19 genome reference was utilized. This comprehensive workflow facilitated the examination and interpretation of genetic information derived from the samples.

## Results

Indications for invasive prenatal diagnosis were as follows: advanced maternal age; fetal ultrasound structural abnormalities, soft markers(such as increased nuchal translucency, mild ventriculomegaly, absent or hypoplastic nasal bone, choroid plexus cyst and echogenic intracardiac foci), previous adverse pregnancies, high risk of serum screening or abnormal non-invasive prenatal testing results, chromosomal abnormalities, consanguineous marriages, a history of adverse exposure during pregnancy; early pregnancy medication history, and abnormal family history. The study aimed to explore the prevalence of ROH and assess the frequency of ROH occurrence on specific chromosomes. In accordance with the consensus guidelines of various countries [5, 6, 16, 18], it is recommended to report ROH and detect UPD in the following cases: the presence of ≥ 5 Mb (located at the end of the chromosome) or ≥ 10 Mb (not at the end of the chromosome) in chromosomes 6, 7, 11, 14, 15, and 20. ROH identified through Chromosomal Microarray Analysis (CMA) was reported following consensus guidelines from various countries.

**Fig. 1:**
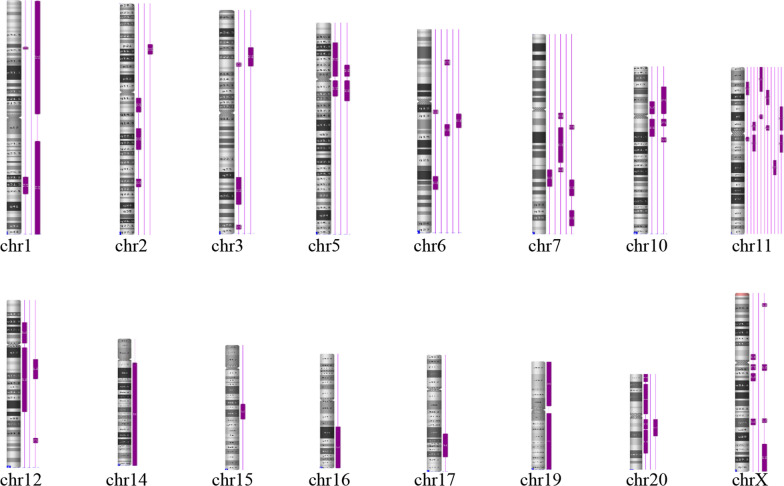
The location of ROH on each chromosome. Each vertical line indicates a fetus, and the thick purple line indicates the location of ROH on the chromosome

The study revealed a prenatal detection rate of ROH ≥ 10 Mb in cases meeting the aforementioned criteria to be 1.8% (33 out of 2007 cases). The most commonly associated chromosome with a single ROH of ≥ 10 Mb was chromosome 11, accounting for 17.1% (6 out of 33 cases). Information about fetuses exhibiting ROH on imprinted chromosomes (6, 7, 11, 14, 15, and 20) is provided in Table 1. Furthermore, Fig. 1 visually represents the distribution of 33 instances of ROH ≥ 10 Mb across chromosomes in the 2007 fetuses examined. This analysis contributes to our understanding of the incidence of ROH on specific chromosomes in the population studied. In Table 2, the indications for invasive prenatal diagnosis are presented for 33 pregnant women with ROH ≥ 10 Mb. Among all diagnostic indicators, the highest proportion is associated with ultrasound abnormalities, followed by a high risk of serum screening.

**Table 1:** SNP-array and karyotype results of 15 fetuses with ROH

No	SNP-array (ROH)	Chromosome	Size (Mb)	Indications	Karyotype	Follow-up
1	arr[hg19] 6q13q14.1(71116171_83031752) × 2 hmz	6	12.0	High risk of serum screening	46,XY,inv(9)(p12q13)	Two-years-old growth and development is normal
2	arr[GRCh37] 6q14.1q15(79854364_89916843) × 2 hmz	6	10.0	Ultrasound soft marker	46,XY	one-year-old growth and development is normal
3	arr[GRCh37]6q22.31q23.2(123304566_134548143) × 2hmz (paternal UPD)	6	11.2	Thickened nuchal fold Agenesis of the corpus callosum; High risk of serum screening; High risk of neural tube defects	46,XY	TOP
4	arr[hg19] 7q31.2q32.1(115333496_128333758) × 2 hmz	7	13.0;12.7	Thickened nuchal translucency	46,XX	A female infant with normal external examination
5	arr[hg19] 7q33q36.3(137994712_157061474) × 2 hmz	7	19.1	First trimester medication history	46,XY	A male infant with normal external examination
6	arr[GRCh37]7q11.23q22.1(74069645_102039696) × 2 hmz (maternal UPD)	7	28.0	Both spouses have thalassemia	CVS karyotype:mos 47,XY, + 7[21]/46,XY,[44]; AF karyotype: 46,XY	TOP
7	arr[hg19] 11p13q13.3(32228901_69217423) × 2 hmz	11	37.0	High risk of serum screening	46,XY	A male infant with normal external examination
8	arr[GRCh37] 11q13.4q14.2(75162918_87295319) × 2 hmz (exclude UPD)	11	12.1	Advanced maternal age	46,XY	A male infant with normal external examination
9	arr[GRCh37] 11p15.1p14.1(18804524_30833779) × 2 hmz (exclude UPD)	11	12.0	Get a COVID-19 vaccine in the first trimester	46,XX	A female infant with normal external examination
10	arr[GRCh37] 11p15.5p15.1(230751_20060445) × 2 hmz	11	19.8	Thickened nuchal fold; ultrasound soft marker	46,XY	9 months old growth and development is normal
11	arr[GRCh37] 11p11.2q13.2(44287149_68239940) × 2 hmz	11	23.9	Both spouses are intellectual disability	46,XY	TOP
12	arr[GRCh37] 11p15.3p14.3(12110677_23047939) × 2 hmz	11	10.9	Thickened nuchal translucency; Pervious adverse pregnancies	46,XY	6 months old growth and development is normal
13	arr[GRCh37]14q11.2q32.33(20520198_107279475) × 2 hmz (paternal UPD)	14	Chromosome 14	Thickened nuchal fold; Agenesis of the corpus callosum	46,XY	TOP
14	arr[hg19] 15q21.1q22.2(48335576_60931242) × 2 hmz (exclude UPD)	15	12.6	Thickened nuchal translucency; Cardiac abnormalities	46,XX	TOP
15	arr[GRCh37] 20q11.21q12(29510307_40604830) × 2 hmz	20	11.1	Fetal bowel echo enhancement, left ventricular intense spot	46,XX	18 months old growth and development is normal

TOP: termination of pregnancy

Thickened nuchal translucency (NT): NT > 3.0 mm; Thickened nuchal fold (NF): NF > 6.0 mm

**Table 2:** Indications for invasive prenatal diagnosis in 33 pregnancies with ROH ≥ 10 Mb

Indications	Patients with ROH
Ultrasound abnormalities	16
High risk of serum screening	8
Pervious adverse pregnancies	7
Advanced maternal age	5
Abnormal NIPT results	5
COVID-19 vaccination during early pregnancy	2
Thalassemia in both parents	2
Parental genetic factors	1
Abnormal karyotypes of parents	1
Pregnancy radiation exposure history	1

NIPT: non-invasive prenatal testing

## Discussion

For decades, karyotype analysis has been considered the gold standard for detecting chromosome aneuploidy during prenatal diagnosis. Karyotype and Chromosomal Microarray Analysis (CMA) are two prenatal diagnostic methods that have been widely used in recent years [10, 21, 23]. As molecular cytogenetic methods have advanced, (CMA has gained prominence and is now suggested as a primary diagnostic test for prenatal assessments. The potential of CMA and the challenges, when compared to conventional karyotyping, are discussed below [19]. However, CMA cannot detect balanced translocations or inversions. Karyotype analyses and SNP-arrays have their own advantages and limitations, and the combination of these methods provides a rigorous diagnosis [10, 23]. Uniparental disomy (UPD) is a significant factor in various diseases, including abnormal fetal development, intellectual disabilities, and developmental delays in children. Approximately 1 in 2000 individuals carry UPD, and the incidence rate of imprinting diseases is relatively high, at approximately 1 in 4000 [20]. ROH in chromosomes can result in homozygous expression of recessive pathogenic genes, leading to increased incidence of recessive genetic diseases.

In this study, out of the 2007 samples with indications for invasive prenatal diagnosis, 33 cases exhibited ROH ≥ 10 Mb. Figure 1 illustrates that chromosome 11 is the most frequently observed chromosome with ROH ≥ 10 Mb, constituting 17.1% (6 cases out of 33). Among all diagnostic indicators, ultrasound abnormalities are associated with the highest proportion, followed by a high risk of serum screening (Table 2). While we have noted this discovery, the precise molecular mechanism behind it remains unclear, necessitating further research. A study by Liu et al. [17] conducted a large-scale investigation on the indication of invasive prenatal diagnosis in absence of heterozygosity cases and found that ultrasound abnormalities accounted for the highest proportion, consistent with our data. Three cases of imprinting diseases caused by UPD were identified using SNP-array technology (Table 1). In case 6, maternal UPD of chromosome 7 was detected through trios-CMA, which involved comparing CMA results between uncultured amniocytes and parental peripheral blood. The karyotype of the chorionic villus sample (CVS showed mosaicism with 47,XY,+7[21]/46,XY[44], while the karyotype of amniocytes was 46,XY. Placental mosaicism of trisomy 7 was confirmed in the prenatal CVS. The presence of 46, XY karyotype in amniocytes may be attributed to trisomy self-rescue [29], in which the extra paternal chromosome 7 is eliminated, resulting in a diploid cell with both chromosomes inherited from the maternal side. When mosaicism for trisomy was observed in the CVS on imprinted chromosomes, consideration should be given to the possibility of confined placental mosaicism. Further chromosomal karyotyping and CMA testing of amniotic fluid samples are required to rule out UPD in diploid cells formed as a result of trisomy rescue. Chromosomal mosaicism might also be associated with the formation of UPD [7]. According to the consensus guidelines [7, 18], maternal UPD of chromosome 7 is associated with a risk of Silver Russell syndrome [8]. The couple chose to undergo termination of pregnancy (TOP) after being informed of the risk. Cases 3 and 13 had abnormal ultrasound soft markers and were found to be at risk of transient neonatal diabetes (paternal UPD of chromosome 6) [11, 15] and Kagami-Ogata syndrome (paternal UPD of chromosome 14) [13]. They chose to undergo TOP after being informed of the risk. Due to the formation mechanisms of UPD, low level or undetected mosaicisms are assumed for a significant number of UPD cases [7]. ROH caused by UPD on chromosomes 6, 7, 11, 14, 15 and 20, may lead to UPD-related disease. When large ROH is detected on chromosomes 6, 7, 11, 14, 15 and 20, UPD verification should be performed, even if the imprinted gene is not in the ROH [5, 18]. In this study, when a ROH of ≥ 10 Mb occurred on a single chromosome, the most frequently involved chromosome was chromosome 11 (17.1%[6/33]). However, another study showed that chromosomes X, 2, and 16 were the most frequently involved [17]. Additionally, Wen et al*.* [27] found that ROH (> 1 Mb) was most frequently observed on chromosomes 8, 2, 6, and 10. It is possible that the sample size was not large enough for the evidence to be conclusive. Indications for ROH cases in this study appeared almost at random. We reported that ROH of ≥ 10 Mb increases the risk of recessive diseases. At the last follow-up evaluation, the children showed no abnormalities. It is possible that the duration of follow-up was not long enough, so follow-up evaluations will continue. Our study had some limitations. While a SNP array analysis can detect isodisomy, detection of heterodisomy requires analysis of parents. However, many parents do not undergo the trios-CMA test due to its cost [22]. Heterodisomy cannot be detected through CMA testing when only fetal CMA is performed. Previous studies have demonstrated cases where heterodisomy leads to imprinting disorders [9, 30]. In this study, ROH ≥ 10 Mb was detected in 33 out of 2007 fetal specimens. However, heterodisomy resulting from imprinting disorders could not be excluded, as CMA testing was not performed on the parents of the remaining 1974 cases. Additionally, the origin of ROH was not identified by parental samples in most cases. Moreover, the data were obtained from a single center, and incomplete follow-up information may have led to an underestimation of late-onset adverse phenotypes. A SNP array can detect large ROH and UPD [12, 25], whilst trios-CMA can effectively detect UPD that cannot be identified by karyotype analysis. This may aid in decision-making regarding pregnancy termination in cases of UPD-related disease.

## Conclusion

CMA has the capability to detect UPD, a capacity beyond the reach of karyotype analysis. Additionally, trios-CMA can further confirm imprinting diseases caused by imprinted genes, thereby aiding in the prevention of such disorders in newborns. The integration of UPD detection by CMA offers a more precise approach to prenatal genetic diagnosis.

## Data Availability

The data that support the findings of this study are available on request from the corresponding author. The data are not publicly available due to privacy or ethical restrictions.

## Abbreviations

CMA: Chromosomal microarray analysis
SNP-array: Single nucleotide polymorphism array
ROH: Regions of homozygosity
UPD: Uniparental disomy

## References

[1] Brun BN, Willer T, Darbro BW (2018). Uniparental disomy unveils a novel recessive mutation in POMT2. Neuromuscul Disord.

[2] Bruno DL, White SM, Ganesamoorthy D (2011). Pathogenic Aberrations revealed exclusively by single nucleotide polymorphism (SNP) genotyping data in 5000 samples tested by molecular karyotyping. J Med Genet.

[3] Carmichael H, Shen Y, Nguyen TT (2013). Whole exome sequencing in a patient with uniparental disomy of chromosome 2 and a complex phenotype. Clin Genet.

[4] Zhang X, Committee for the Prevention and Control of Birth Defect Chinese Association of Preventive Medicine (2021). Guidelines for the interpretation of fetal chromosomal karyotyping analysis. Zhonghua Yi Xue Yi Chuan Xue Za Zhi.

[5] Dawson AJ, Chernos J, Mcgowan-Jordan J (2011). CCMG guidelines: prenatal and postnatal diagnostic testing for uniparental disomy. Clin Genet.

[6] Del Gaudio D, Shinawi M, Astbury C (2020). Diagnostic testing for uniparental disomy: a points to consider statement from the American College of Medical Genetics and Genomics (ACMG). Genet Med.

[7] Eggermann T, Soellner L, Buiting K (2015). Mosaicism and uniparental disomy in prenatal diagnosis. Trends Mol Med.

[8] Eggermann T, Spengler S, Gogiel M (2012). Epigenetic and genetic diagnosis of Silver-Russell syndrome. Expert Rev Mol Diagn.

[9] Eggermann T, Wollmann HA, Kuner R (1997). Molecular studies in 37 Silver-Russell syndrome patients: frequency and etiology of uniparental disomy. Hum Genet.

[10] Hao M, Li L, Zhang H (2020). The difference between karyotype analysis and chromosome microarray for mosaicism of aneuploid chromosomes in prenatal diagnosis. J Clin Lab Anal.

[11] Hermann R, Laine AP, Johansson C (2000). Transient but not permanent neonatal diabetes mellitus is associated with paternal uniparental isodisomy of chromosome 6. Pediatrics.

[12] Hoppman N, Rumilla K, Lauer E (2018). Patterns of homozygosity in patients with uniparental disomy: detection rate and suggested reporting thresholds for SNP microarrays. Genet Med.

[13] Kagami M, Kato F, Matsubara K (2012). Relative frequency of underlying genetic causes for the development of UPD(14)pat-like phenotype. Eur J Hum Genet.

[14] King DA, Fitzgerald TW, Miller R (2014). A novel method for detecting uniparental disomy from trio genotypes identifies a significant excess in children with developmental disorders. Genome Res.

[15] Kotzot D (2008). Prenatal testing for uniparental disomy: indications and clinical relevance. Ultrasound Obstet Gynecol.

[16] Lin S, Liu W, Guo L (2022). A consensus on prenatal diagnosis and genetic counseling for chromosomal mosaicism. Zhonghua Yi Xue Yi Chuan Xue Za Zhi.

[17] Liu J, He Z, Lin S (2021). Absence of heterozygosity detected by single-nucleotide polymorphism array in prenatal diagnosis. Ultrasound Obstet Gynecol.

[18] Liu W, Lu J, Zhang J (2020). A consensus recommendation for the interpretation and reporting of copy number variation and regions of homozygosity in prenatal genetic diagnosis. Zhonghua Yi Xue Yi Chuan Xue Za Zhi.

[19] Liu X, Liu S, Wang H (2022). Potentials and challenges of chromosomal microarray analysis in prenatal diagnosis. Front Genet.

[20] Nakka P, Smith SP, O’Donnell-Luria AH (2019). Characterization of prevalence and health consequences of uniparental disomy in four million individuals from the general population. Am J Hum Genet.

[21] Shi Y, Ma J, Xue Y (2019). The assessment of combined karyotype analysis and chromosomal microarray in pregnant women of advanced maternal age: a multicenter study. Ann Transl Med.

[22] Sinkey RG, Odibo AO (2016). Cost-effectiveness of old and new technologies for aneuploidy screening. Clin Lab Med.

[23] Sun W, Su J, Liu T (2022). Comparison of performance of two prenatal diagnostic techniques for the detection of chromosomal mosaicisms in amniocytes. Zhonghua Yi Xue Yi Chuan Xue Za Zhi.

[24] Vermeesch JR, Fiegler H, De Leeuw N (2007). Guidelines for molecular karyotyping in constitutional genetic diagnosis. Eur J Hum Genet.

[25] Wang JC, Ross L, Mahon LW (2015). Regions of homozygosity identified by oligonucleotide SNP arrays: evaluating the incidence and clinical utility. Eur J Hum Genet.

[26] Wapner J, Martin K, Levy Z (2012). Chromosomal microarray versus karyotyping for prenatal diagnosis. N Engl J Med.

[27] Wen J, Comerford K, Xu Z (2019). Analytical validation and chromosomal distribution of regions of homozygosity by oligonucleotide array comparative genomic hybridization from normal prenatal and postnatal case series. Mol Cytogenet.

[28] Wiszniewska J, Bi W, Shaw C (2014). Combined array CGH plus SNP genome analyses in a single assay for optimized clinical testing. Eur J Hum Genet.

[29] Yamazawa K, Ogata T, Ferguson-Smith AC (2010). Uniparental disomy and human disease: an overview. Am J Med Genet C Semin Med Genet.

[30] Zhang L, Liu X, Zhao Y (2022). Genetic subtypes and phenotypic characteristics of 110 patients with Prader-Willi syndrome. Ital J Pediatr.

